# Toll-Like Receptor Signaling Pathways

**DOI:** 10.3389/fimmu.2014.00461

**Published:** 2014-09-25

**Authors:** Takumi Kawasaki, Taro Kawai

**Affiliations:** ^1^Laboratory of Molecular Immunobiology, Graduate School of Biological Sciences, Nara Institute of Science and Technology, Ikoma, Japan

**Keywords:** TLRs, signal transduction, NF-κB, IRFs, adaptors

## Abstract

Toll-like receptors (TLRs) play crucial roles in the innate immune system by recognizing pathogen-associated molecular patterns derived from various microbes. TLRs signal through the recruitment of specific adaptor molecules, leading to activation of the transcription factors NF-κB and IRFs, which dictate the outcome of innate immune responses. During the past decade, the precise mechanisms underlying TLR signaling have been clarified by various approaches involving genetic, biochemical, structural, cell biological, and bioinformatics studies. TLR signaling appears to be divergent and to play important roles in many aspects of the innate immune responses to given pathogens. In this review, we describe recent progress in our understanding of TLR signaling regulation and its contributions to host defense.

## Introduction

The innate immune system employs germline-encoded pattern-recognition receptors (PRRs) for the initial detection of microbes. PRRs recognize microbe-specific molecular signatures known as pathogen-associated molecular patterns (PAMPs) and self-derived molecules derived from damaged cells, referred as damage-associated molecules patterns (DAMPs). PRRs activate downstream signaling pathways that lead to the induction of innate immune responses by producing inflammatory cytokines, type I interferon (IFN), and other mediators. These processes not only trigger immediate host defensive responses such as inflammation, but also prime and orchestrate antigen-specific adaptive immune responses (1). These responses are essential for the clearance of infecting microbes as well as crucial for the consequent instruction of antigen-specific adaptive immune responses.

Mammals have several distinct classes of PRRs including Toll-like receptors (TLRs), RIG-I-like receptors (RLRs), Nod-like receptors (NLRs), AIM2-like receptors (ALRs), C-type lectin receptors (CLRs), and intracellular DNA sensors such as cGAS (2, 3). Among these, TLRs were the first to be identified, and are the best characterized. The TLR family comprises 10 members (TLR1–TLR10) in human and 12 (TLR1–TLR9, TLR11–TLR13) in mouse. TLRs localize to the cell surface or to intracellular compartments such as the ER, endosome, lysosome, or endolysosome, and they recognize distinct or overlapping PAMPs such as lipid, lipoprotein, protein, and nucleic acid. Each TLR is composed of an ectodomain with leucine-rich repeats (LRRs) that mediate PAMPs recognition, a transmembrane domain, and a cytoplasmic Toll/IL-1 receptor (TIR) domain that initiates downstream signaling. The ectodomain displays a horseshoe-like structure, and TLRs interact with their respective PAMPs or DAMPs as a homo- or heterodimer along with a co-receptor or accessory molecule (4). Upon PAMPs and DAMPs recognition, TLRs recruit TIR domain-containing adaptor proteins such as MyD88 and TRIF, which initiate signal transduction pathways that culminate in the activation of NF-κB, IRFs, or MAP kinases to regulate the expression of cytokines, chemokines, and type I IFNs that ultimately protect the host from microbial infection. Recent studies have revealed that proper cellular localization of TLRs is important in the regulation of the signaling, and that cell type-specific signaling downstream of TLRs determines particular innate immune responses. Here, we summarize recent progress on TLR signaling pathways and their contributions to host defense responses.

## PAMP Recognition by TLRs

TLRs are expressed in innate immune cells such as dendritic cells (DCs) and macrophages as well as non-immune cells such as fibroblast cells and epithelial cells. TLRs are largely classified into two subfamilies based on their localization, cell surface TLRs and intracellular TLRs. Cell surface TLRs include TLR1, TLR2, TLR4, TLR5, TLR6, and TLR10, whereas intracellular TLRs are localized in the endosome and include TLR3, TLR7, TLR8, TLR9, TLR11, TLR12, and TLR13 (5, 6).

Cell surface TLRs mainly recognize microbial membrane components such as lipids, lipoproteins, and proteins. TLR4 recognizes bacterial lipopolysaccharide (LPS). TLR2 along with TLR1 or TLR6 recognizes a wide variety of PAMPs including lipoproteins, peptidoglycans, lipotechoic acids, zymosan, mannan, and tGPI-mucin (5). TLR5 recognizes bacterial flagellin (2). TLR10 is pseudogene in mouse due to an insertion of a stop codon, but human TLR10 collaborates with TLR2 to recognize ligands from listeria (7). TLR10 can also sense influenza A virus infection (8).

Intracellular TLRs recognize nucleic acids derived from bacteria and viruses, and also recognize self-nucleic acids in disease conditions such as autoimmunity (9). TLR3 recognizes viral double-stranded RNA (dsRNA), small interfering RNAs, and self-RNAs derived from damaged cells (10–12). TLR7 is predominantly expressed in plasmacytoid DCs (pDCs) and recognizes single-stranded (ss)RNA from viruses. It also recognizes RNA from streptococcus B bacteria in conventional DCs (cDCs) (13). Human TLR8 responds to viral and bacterial RNA (14). Structural analysis revealed that unstimulated human TLR8 exists as a preformed dimer, and although the Z-loop between LRR14 and LRR15 is cleaved, the N- and C-terminal halves remain associated with each other and participate in ligand recognition and dimerization. Ligand binding induces reorganization of the dimer to bring the two C termini into close proximity (15). TLR13 recognizes bacterial 23S rRNA (16–18) and unknown components of vesicular stomatitis virus (19). TLR9 recognizes bacterial and viral DNA that is rich in unmethylated CpG-DNA motifs; it also recognizes hemozoin, an insoluble crystalline byproduct generated by *Plasmodium falciparum* during the process of detoxification after host hemoglobin is digested (20). TLR11 is localized in the endolysosome and recognizes flagellin (21) or an unknown proteinaceous component of uropathogenic *Escherichia coli* (UPEC) as well as a profilin-like molecule derived from *Toxoplasma gondii* (22). TLR12 is predominantly expressed in myeloid cells and is highly similar to TLR11 and recognizes profilin from *T. gondii* (23). TLR12 functions either as a homodimer or a heterodimer with TLR11 (24, 25).

## Trafficking of TLRs

All TLRs are synthesized in the ER, traffic to the Golgi, and are recruited to the cell surface or to intracellular compartments such as endosomes. Intracellular localization of TLRs is thought to be critical for ligand recognition as well as for preventing TLRs from coming into contact with self-nucleic acids, which could cause autoimmunity (26–29). The multi-pass transmembrane protein UNC93B1 controls the trafficking of intracellular TLRs from the ER to endosomes. Interestingly, UNC93B1 regulates excessive TLR7 activation by employing TLR9 to counteract TLR7. This was demonstrated by experiments in mice harboring an amino acid substitution (D34A) in UNC93B1, which exhibit a TLR7-hyperreactive and TLR9-hyporeactive phenotype associated with TLR7-dependent systemic lethal inflammation. Thus, a optimizing the balance between TLR7 and TLR9 is a potential mechanism for regulating autoimmunity (30). TLR trafficking is also controlled by the ER-resident protein PRAT4A, which regulates the exit of TLR1, TLR2, TLR4, TLR7, and TLR9 from the ER and their trafficking to the plasma membrane and endososmes (31). gp96, a member of the ER-resident heat-shock protein 90 family, functions as a general chaperone for most TLRs, including cell surface TLR1, TLR2, TLR4, and TLR5 and intracellular TLR7 and TLR9 (32).

In the endosome, nucleic acid-sensing TLRs undergo proteolytic cleavage by cathepsins B, S, L, H, and K and asparginyl endopeptidase to attain a functional form that mediates ligand recognition and initiates signaling (33–35). However, the N-terminal region of TLR9 is required for CpG-DNA recognition and binding (36). Interestingly, a recent study suggests that the N-terminal cleaved fragment (TLR9N) remains associated with truncated TLR9 (TLR9C) to form a complex, which acts as a functional DNA sensor (37).

## Contribution of TIR Domain-Containing Adaptors to TLR Signaling

**Figure 1:**
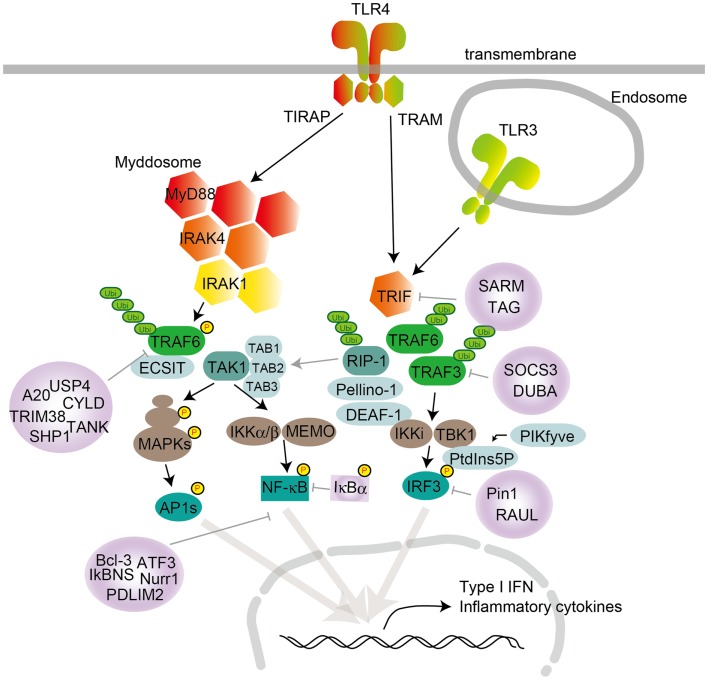
**TLR signaling in cDCs, macrophages, and MEFs**. TLR4 localize to the cell surface, and TLR3 localize in the endosome compartment. Homo- or heterodimer formation initiates signaling to the two major downstream adaptor proteins, MyD88 and TRIF. TIRAP conducts the signal from TLR4 to MyD88, and TRAM mediates the signal from TLR4 to TRIF. TLR engagement induces formation of the Myddosome, which is based on MyD88 and also contains IRAK1 and IRAK4. IRAK1 activation induces TRAF6 activation following K63-linked polyubiquitination on TRAF6 itself and TAK1. TAK1 activation leads to the activation of IKK complex-NF-κB and MAPKs. MAPK activation leads to AP1s transcription factor activation. TRAF6 promotes ECSIT ubiquitination, resulting in increased mitochondrial and cellular ROS generation. TLR engagement also induces TRIF activation following TRAF6 and TRAF3 recruitment. TRAF6 recruits RIP-1, which activates the TAK1 complex following MAPK activation. RIP-1 activation regulates ubiquitination by Pellino-1. Pellino-1 regulates IRF3 activation by binding to DEAF-1. TRAF3 recruits TBK1 and IKKi for IRF3 phosphorylation. PtdIns5P from PIKfyve facilitates complex formation between TBK1 and IRF3. Several negative regulators modulate TLR signaling, by inhibiting either signaling complex formation or ubiquitination. MyD88 is suppressed by ST2825, NRDP-1, SOCS1, and Cbl-b; TRIF is suppressed by SARM and TAG; TRAF3 is suppressed by SOCS3 and DUBA; and TRAF6 is suppressed by A20, USP4, CYLD, TANK, TRIM38, and SHP. NF-κB is suppressed by Bcl-3, IκBNS, Nurr1, ATF3, and PDLIM2, while IRF3 activation is negatively regulated by Pin1 and RAUL.

Individual TLRs differentially recruit members of a set of TIR domain-containing adaptors such as MyD88, TRIF, TIRAP/MAL, or TRAM. MyD88 is utilized by all TLRs and activates NF-κB and MAPKs for the induction of inflammatory cytokine genes. TIRAP is a sorting adaptor that recruits MyD88 to cell surface TLRs such as TLR2 and TLR4 (Figure 1). However, a recent study demonstrated that TIRAP also participates in signaling through endosomal TLRs such as TLR9. The lipid-binding domain of TIRAP binds to PI(4,5)P_2_ at the plasma membrane and to PI(3)P on endosomes, which mediates the formation of functional TLR4 and TLR9 signaling complexes at their respective sites. Thus, TIRAP associates with both cell surface and endosomal TLRs by binding to different lipids (38). However, a high concentration of TLR9 agonists activates cells in the absence of TIRAP, suggesting that TIRAP is required for TLR9 signaling in natural situations such as HSV-1 infection (39).

TRIF is recruited to TLR3 and TLR4 and promotes an alternative pathway that leads to the activation of IRF3, NF-κB, and MAPKs for induction of type I IFN and inflammatory cytokine genes. TRAM is selectively recruited to TLR4 but not TLR3 to link between TRIF and TLR4. TLR3 directly interacts with TRIF, and this interaction requires phosphorylation of the two tyrosine residues in the cytoplasmic domain of TLR3 by the epidermal growth factor ErbB1 and Btk (40, 41). Collectively, depending on the adaptor usage, TLR signaling is largely divided into two pathways: the MyD88-dependent and TRIF-dependent pathways.

## MyD88-Dependent Pathway

After TLR engagement, MyD88 forms a complex with IRAK kinase family members, referred to as the Myddosome (Figure 1) (42). During Myddosome formation, IRAK4 activates IRAK1, which is then autophosphorylated at several sites (43) and released from MyD88 (44). IRAK1 associates with the RING-domain E3 ubiquitin ligase TRAF6. TRAF6, along with ubiquitin-conjugating enzyme UBC13 and UEV1A, promotes K63-linked polyubiquitination of both TRAF6 itself and the TAK1 protein kinase complex. TAK1 is a member of the MAPKKK family and forms a complex with the regulatory subunits TAB1, TAB2, and TAB3, which interact with polyubiquitin chains generated by TRAF6 to drive TAK1 activation (45, 46). Although the mechanisms of TAK1 activation within this complex remain unclear, K63-linked ubiquitination or close proximity-dependent transphosphorylation may be responsible for TAK1 activation. TAK1 then activates two different pathways that lead to activation of the IKK complex-NF-κB pathway and -MAPK pathway. The IKK complex is composed of the catalytic subunits IKKα and IKKβ and the regulatory subunit NEMO (also called IKKγ). TAK1 binds to the IKK complex through ubiquitin chains, which allows it to phosphorylate and activate IKKβ. The IKK complex phosphorylates the NF-κB inhibitory protein IκBα, which undergoes proteasome degradation, allowing NF-κB to translocate into the nucleus to induce proinflammatory gene expression. TAK1 activation also results in activation of MAPK family members such as ERK1/2, p38 and JNK, which mediates activation of AP-1 family transcription factors or stabilization of mRNA to regulate inflammatory responses (2, 5).

TAK1 deficiency in mouse embryonic fibroblast cells (MEFs) reduces phosphorylation of IKKs, p38, and JNK after LPS stimulation. However, TLR4-mediated IKK, p38, and JNK activation and cytokine induction are increased in neutrophils derived from TAK1-deficient mice, suggesting a cell type-specific role for TAK1 in TLR signaling (47). Furthermore, the physiological roles of TAB proteins in TLR signaling also remain controversial: TAB1- or TAB2-deficient mice do not show any abnormality in TLR signaling pathways (48), and mice doubly deficient for TAB2 and TAB3 also exhibit normal cytokine production after TLR simulation in MEFs and macrophages (49). TAB family proteins may therefore compensate for each other in TLR signaling.

TLR2 and TLR4 ligations in macrophages increase the production of mitochondrial ROS for bactericidal action and recruit mitochondria to phagosomes (50). TRAF6 is translocated to mitochondria following bacterial infection, where it interacts with ECSIT. TRAF6 promotes ECSIT ubiquitination, resulting in increased mitochondrial and cellular ROS generation.

## TRIF-Dependent Pathway

TRIF interacts with TRAF6 and TRAF3. TRAF6 recruits the kinase RIP-1, which in turn interacts with and activates the TAK1 complex, leading to activation of NF-κB and MAPKs and induction of inflammatory cytokines (Figure 1). In contrast, TRAF3 recruits the IKK-related kinases TBK1 and IKKi along with NEMO for IRF3 phosphorylation. Subsequently, IRF3 forms a dimer and translocates into the nucleus from the cytoplasm, where it induces the expression of type I IFN genes (2, 5).

The Pellino family E3 ubiquitin ligases are implicated in TLR signaling (51). Pellino-1-deficient mice display impaired TRIF-dependent NF-κB activation and cytokine production (52). Pellino-1 is phosphorylated by TBK1/IKKi and thereby facilitates ubiquitination of RIP-1, suggesting that Pellino-1 mediates TRIF-dependent NF-κB activation by recruiting RIP-1. Furthermore, Pellino-1 regulates IRF3 activation by binding to DEAF-1, a transcription factor that facilitates binding of IRF3 to the IFNβ promoter (51).

Recently, IRF3 activation was demonstrated to be regulated by an inositol lipid, PtdIns5P. PtdIns5P binds to both IRF3 and TBK1, and thus facilitates complex formation between TBK1 and IRF3. The accessibility of TBK1 to IRF3 mediated by PtdIns5P likely causes IRF3 phosphorylation in a closely proximal manner. Furthermore, PIKfyve was identified as a kinase responsible for production of PtdIns5P during virus infection (53).

## Balanced Activation Between MyD88- and TRIF-Dependent Pathways

TLR4 activates both the MyD88-dependent and TRIF-dependent pathways. Activation of these pathways is controlled by several molecules to induce appropriate responses. Balanced production of inflammatory cytokines and type I IFN may be important for controlling tumor cell growth and autoimmune diseases.

TRAF3 was shown to be incorporated into the MyD88 complex as well as the TRIF complex in TLR4 signaling. TRAF3 within the MyD88 complex is then degraded, which causes TAK1 activation. Thus, in addition its role in promoting TRIF-dependent pathway activation, TRAF3 has a role in inhibiting the MyD88-dependent pathway. NRDP-1, an E3 ubiquitin ligase, binds and ubiquitinates MyD88 and TBK1, inducing the degradation of MyD88 and augmenting the activation of TBK1, which attenuates inflammatory cytokine production and induces preferential type I IFN production, respectively (54).

MHC class II molecules that are localized in endosomes in antigen-presenting cells interact with the tyrosine kinase Btk via the costimulatory molecule CD40 and maintain Btk activation. Activated Btk interacts with MyD88 and TRIF to promote the activation of the MyD88-dependent and TRIF-dependent pathways and thus to enhance production of inflammatory cytokines and type I IFNs, respectively (55).

## TLR7 and TLR9 Signaling in Plasmacytoid DCs

**Figure 2:**
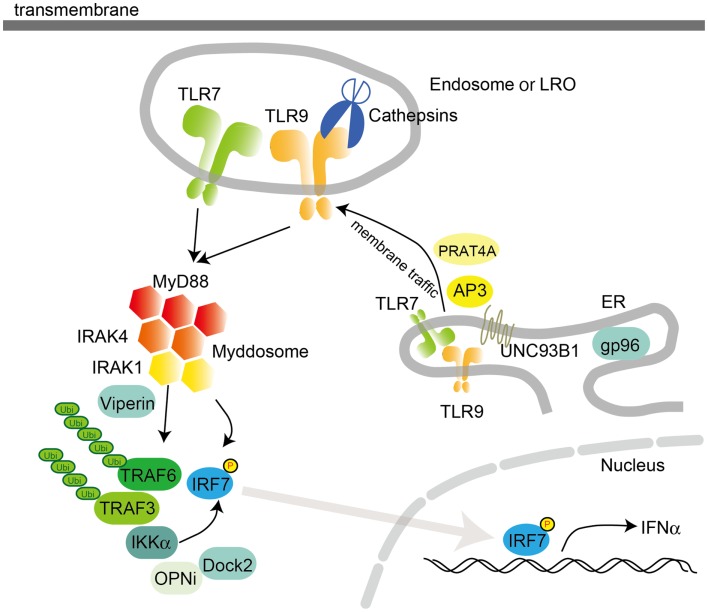
**Intracellular TLR signaling and trafficking in pDCs**. Activation of TLR7 or TLR9 in pDCs recruits MyD88 following IRAK4 recruitment. The MyD88 complex also contains TRAF3, TRAF6, IRAK4, IRAK1, IKKα, OPNi, and Dock2. MyD88 directly or indirectly recruits IRF7 to be phosphorylated by IKKα and/or IRAK1. Localization of TLR7 and 9 is controlled by UNC93B1, PRAT4A, and AP3, which traffic TLRs from the ER to the endosome or the lysosome-related organelle (LRO). In the endosome, TLRs are converted to their mature forms by cathepsins, which cleave LRRs in the ectodomain. pDCs utilize a distinct signaling pathway from that in cDCs or macrophages to induce the synthesis of large amount of type I IFNs. gp96, a member of the ER-resident heat-shock protein 90 family, functions as a general chaperone for most TLRs.

Plasmacytoid DCs are a subset of DCs with the capacity to secrete vast amounts of type I IFN in response to viral infection (Figure 2) (2, 5). In pDCs, TLR7 and TLR9 serve as primary sensors for RNA and DNA viruses, respectively. Interestingly, the production of type I IFN by pDCs relies on a complex containing MyD88 and IRF7. This complex also contains TRAF3, TRAF6, IRAK4, IRAK1, IKKα, OPNi, and Dock2 (56, 57). Within this complex, IRF7 is phosphorylated by IRAK1 and/or IKKα and translocates into the nucleus to regulate the expression of type I IFN. Moreover, MyD88-IRAK4-TRAF6 complex drives NF-κB-dependent inflammatory cytokine induction. The signaling complex containing MyD88-IRAK1-TRAF6-IRF7 is formed within lipid bodies by the IFN-inducible Viperin, which activates IRAK1 by lysine 63-linked ubiquitination (58). It is notable that TLR9 signals through different cellular compartments that induce either MyD88-IRF7-dependent type I IFN or MyD88- NF-κB -dependent inflammatory cytokines (59). TLR9 initially traffics to VAMP3-positive early endosomes after CpG-DNA stimulation, where it triggers MyD88-IRAK4-TRAF6-dependent NF-κB activation. TLR9 then traffics to LAMP2-positive lysosome-related organelles (LROs), where it incorporates TRAF3 to activate IRF7 and induce type I IFN (Figure 2). AP3 has been shown to bind to TLR9 and control the trafficking of TLR9 to LROs, and is required for type I IFN induction (28). However, AP3 is not required for TLR9-dependent type I IFN induction triggered by DNA-antibody immune complexes (ICs) in pDCs. The intracellular compartment initiating type I IFN induction by DNA-antibody ICs is regulated by the autophagy pathway (60). Thus, pDCs have diverse cargoes for ligand recognition and triggering downstream signaling pathways.

## Other IRFs in TLR Signaling

In addition to IRF3 and IRF7, several other IRFs participate in TLR signaling. IRF1 interacts with MyD88 and contributes to TLR9-mediated cytokine production in the presence of IFNγ (61), while IRF5 is involved in the MyD88-dependent signaling pathway for inducing inflammatory cytokine production (62). IRF8 was proposed to be essential for TLR9-MyD88-dependent activation of NF-κB in pDCs (63). However, a subsequent analysis of IRF8-deficient mice demonstrated that IRF8 is involved in the second phase of feedback type I IFN production after treatment of DCs with TLR agonists (64).

## Activation of TLR Signaling by Co-Receptors

Recent studies have identified several transmembrane molecules that modulate TLR signaling pathways. CD14, a glycophosphatidylinositol-anchored protein, is a co-receptor with TLR4 and MD-2 for LPS recognition. It induces ITAM-mediated Syk- and PLCγ2-dependent endocytosis to promote TLR4 internalization into endosomes for activation of TRIF-dependent signaling (65). CD14 is also required for TLR7- and TLR9-dependent induction of proinflammatory cytokines (66).

CD36, a protein in the class B scavenger receptor family, acts as a co-receptor for oxidized low-density lipoprotein (LDL) and amyloid-β peptide. Ligand recognition induces the assembly of TLR4/TLR6 heterodimers through Src kinases and consequent sterile inflammation, by inducing inflammatory cytokines and ROS and priming NLRP3 inflammsome activation (67, 68).

## Negative Regulators

TLR signaling is negatively regulated by a number of molecules through various mechanisms to prevent or terminate the excessive immune responses that lead to detrimental consequences associated with autoimmunity and inflammatory diseases. Negative regulators target each of the key molecules in TLR signaling (Figure 1). Activation of the MyD88-dependent pathway is suppressed by ST2825, SOCS1, and Cbl-b, and activation of the TRIF-dependent pathway is suppressed by SARM and TAG (69, 70). These molecules associate with MyD88 or TRIF to prevent them from binding to TLRs or downstream molecules. TRAF3 activation is negatively regulated by SOCS3 and DUBA (71). TRAF6 is targeted by a number of inhibitory molecules such as A20, USP4, CYLD, TANK, TRIM38, and SHP (72–74). TAK1 activation is inhibited by TRIM30α and A20 (75). In addition to these signaling molecules, the transcription factor NF-κB is suppressed by Bcl-3, IκBNS, Nurr1, ATF3, and PDLIM2, while IRF3 activation is negatively regulated by Pin1 and RAUL (76). The stability of mRNAs encoding signaling molecules is regulated by miRNAs such as miR-146a, miR-199a, miR-155, miR-126, miR-21, miR-29, miR-148/152, and miR-466l (74). In addition to the stability of mRNAs for signaling molecules, stability of mRNA for cytokines is regulated by Regnase-1 and TTP (5, 74).

## Concluding Remarks

During the past decade, tremendous progress has been made in our understanding of TLR signaling pathways. After genetic studies revealed the contribution of TIR domain-containing adaptor usage, cell biological and biochemical approaches have highlighted the importance of cellular localization of these adaptors in the regulation of downstream signaling. Moreover, numerous reports have demonstrated that TLR trafficking, TLR cleavage, and protein modification of signaling molecules such as ubiquitination and phosphorylation play important roles in the activation of TLR signaling. On the other hand, negative regulators of TLR signaling have been discovered, and their importance in preventing autoimmune and inflammatory diseases is recognized. More recently, much effort has been focused on identifying molecules that are involved in innate immunity through an integrated approach. Indeed, by combining transcriptomics, genetic/chemical perturbations and phosphoproteomics, Polo-like kinases (Plks) 2 and 4 have been found to regulate antiviral responses downstream of TRIF and MyD88 signaling (77). mRNA stability has also attracted attention because it is an important mechanism to regulate TLR-dependent inflammation. For example, the RNase Regnase-1 interacts with IL-6 and IL-12p40 mRNA and degrades them. Regnase-1-deficient macrophages produce large amounts of cytokines after treatment with various TLR ligands, and Regnase-1-deficient mice show elevated autoantibody production (78). Furthermore, it is notable that PAMP variants may activate distinct signaling pathways although they are recognized by the same PRRs. For example, LPS variant such as smooth or rough type activates either MyD88-dependent or TRIF-dependent pathway. These findings suggest that host makes a distinction between different types of LPS-containing bacteria by activating distinct signaling pathways (79).

Although PAMP recognition by TLRs is crucial for host defense responses to pathogen infection, aberrant activation of TLR signaling by PAMPs, mutations of TLR signaling molecules, and DAMPs-mediated TLRs signaling activation are responsible for the development of several diseases such as autoimmune, chronic inflammatory, and allergic diseases. Moreover, a link between cancer and TLRs has been proposed. The innate immune activation that caused after anti-cancer drug treatment is reportedly critical for cancer elimination through TLR-mediated recognition of endogenous molecules released from dying cancer cells (80). On the contrary, mutations in molecules involved in TLR signaling are associated with cancer development. Certain types of diffuse large B-cell lymphoma acquire oncogenic ability through MyD88 mutation and show aberrant activation of NF-κB, JAK and STAT3 (81). A mutation in A20, which is a negative regulator of TLR signaling, is also associated with B-cell lymphoma development (82, 83). Furthermore, it has been suggested that TBK1 functions as a negative regulator of cell growth in lung cancer (84). In summary, further elucidation of TLR signaling pathways should eventually allow us to manipulate them in strategies to treat various infectious and autoimmune diseases that are intimately associated with innate immune signaling, as well as cancer.

## Conflict of Interest Statement

The authors declare that the research was conducted in the absence of any commercial or financial relationships that could be construed as a potential conflict of interest.
